# Game-Based Social-Emotional Learning for Youth: School-Based Qualitative Analysis of Brain Agents

**DOI:** 10.2196/67550

**Published:** 2025-07-24

**Authors:** Elizabeth Liverman, David Antognoli, Cordelia Elaiho, Madison McGuire, Abbey Stoltenburg, Angel Navarrete, Garrett Bates, Thomas Chelius, Constance Gundacker, Paula Lumelsky, Brandon Currie, John Meurer Sr

**Affiliations:** 1 School of Medicine, Medical College of Wisconsin, 8701 Watertown Plank Road, Milwaukee, WI, 53226, United States, 1 4145100375; 2 School of Design, Columbia College Chicago, Chicago, IL, United States; 3 Night City, Chicago, IL, United States; 4 School of Medicine, Southern Illinois University, Springfield, IL, United States; 5 School of Graduate Studies, Institute for Health & Humanity, Medical College of Wisconsin, Milwaukee, WI, United States; 6 Department of Pediatrics, Medical College of Wisconsin, Milwaukee, WI, United States; 7 STRYV365, Milwaukee, WI, 53212, United States

**Keywords:** trauma-informed programming, social-emotional learning, game-based learning, adolescent mental health, qualitative research, educational video games

## Abstract

**Background:**

Adverse childhood experiences such as violence, substance use, and family disruption disproportionately affect youth in urban communities, increasing the risk of emotional and behavioral challenges. Social-emotional learning (SEL) and trauma-informed programming are effective strategies for mitigating these effects, fostering resilience, and promoting mental well-being. Game-based learning is a promising, engaging method for delivering SEL content. STRYV365 developed *Brain Agents*, a trauma-informed, game-based SEL intervention aimed at improving emotional regulation, coping strategies, and interpersonal skills among students in grades 5 through 9.

**Objective:**

This study explored students’ experiences with and perceptions of *Brain Agents*, evaluating its effectiveness in fostering SEL skills and resilience across 4 diverse urban schools in Milwaukee, Wisconsin.

**Methods:**

A cluster-randomized, incomplete block factorial crossover design was implemented from 2022-2024. Of 1626 eligible students, 329 (20%) had caregiver consent and student assent. Among these, 180 students in grades 5‐9 played *Brain Agents* at school over 4‐5 weeks, for an average of 10 sessions and 23 minutes per session. SEL-related outcomes were assessed using surveys, focus groups, and interviews. Qualitative data were analyzed using Dedoose software, with thematic coding conducted by multiple coders to ensure reliability.

**Results:**

Student demographics included 189/321 (58.9%) Black, 112/321 (34.9%) White, and 221/321 (68.8%) from economically disadvantaged backgrounds. Baseline surveys of 277 children revealed that 202 (72.9%) of students had experienced the death of someone close, 147 (53.1%) had a close contact incarcerated, and 39 (14.1%) reported feeling nervous or anxious daily. Strengths included 230 (83.0%) students reporting life satisfaction and 183 (66.1%) able to calm down when upset. Game performance data from 328 students indicated varying levels of achievement, with a median of 3 (IQR 1.5-4) missions completed, 4 (IQR 2-6) stars earned, 8 positive energies collected, and 2 (IQR 1-2.5) crew members rescued. Grades 7‐8 had the highest engagement, while grade 9 students had the lowest participation. Qualitative analysis from 62 participants identified 8 core themes: qualities of most pride, neighborhood relationships, challenges in life, emotions associated with loss of control, coping strategies, future goals, experiences with *Brain Agents,* and suggestions to improve the game. Students most frequently cited anger as a cause of emotional dysregulation and named coping strategies such as self-calming, asking for help, and perseverance. Feedback on *Brain Agents* highlighted improved focus, emotional control, and critical thinking, with younger students more positively engaged. Suggested improvements included better graphics, more customization, and cooperative play.

**Conclusions:**

*Brain Agents* was positively received by students, particularly those in earlier grades, and demonstrated potential as an effective trauma-informed SEL tool. The findings support the role of game-based interventions in enhancing resilience and emotional intelligence among youth exposed to adversity. Broader implementation may extend benefits to diverse student populations and settings.

## Introduction

### Adverse Childhood Experiences

Child and adolescent health and well-being are influenced by various factors, including early life experiences and educational opportunities. One way to measure trauma in early life is through adverse childhood experiences (ACEs), which include familial incarceration, drug use in the home, and witnessing or experiencing violence [1]. Youth exposure to trauma is linked to poor health, school absenteeism, and behavioral issues [2]. The COVID-19 pandemic is a recent ACE that disrupted typical childhood interactions, causing significant hardships for many children [3]. The COVID-19 pandemic saw a rise in gaming as a coping mechanism, reflecting the role of games in promoting relaxation, emotional regulation, and social connection during periods of stress [4]. Despite indicators of positive mental health in most children, such as curiosity, persistence, and self-control, depression and anxiety have increased over the past 2 decades. For adolescents, depression, substance use, and suicide are significant concerns [1]. Given the prevalence of ACEs and rising concerns for student mental health, programs to support student resilience and behavioral and emotional regulation have become essential.

### Trauma-Informed Programming

Trauma-informed programming is one method used to address these issues. This approach sensitizes educators to trauma while improving school engagement, reducing behavioral problems, and enhancing students’ social and emotional skills [5]. The goal of trauma-informed programming is to reduce stress, increase coping abilities, and decrease impulsive behavior in youth [6]. Another complementary approach is social-emotional learning (SEL), which focuses on self-awareness, self-management, and social mindfulness to help youth make responsible decisions, achieve their goals, and build relationships [7]. SEL is closely tied to emotional intelligence, the ability to monitor and manage one’s own and others’ emotions and use this information to guide thinking and actions [8]. The ability model of emotional intelligence focuses on the capacity to accurately perceive, understand, and manage emotions—skills that are essential for building resilience and social-emotional competence in youth [9]. School-based SEL programs can lead to positive attitudes toward self and others, positive behavior, academic success, and mental wellness [10]. Research has shown that video games can support emotion regulation, a core component of SEL [11]. This evidence reinforces the use of a game-based tool to help students identify and manage emotions.

### Game-Based Learning

Game-based learning is an innovative way to integrate trauma-informed programming and SEL into school curricula. This strategy applies game elements such as scoring, challenge, narrative, role-playing, and progression to motivate and engage players in educational content where they can learn by doing [12]. While excessive use of video games can have negative health and behavior implications, video games also offer many benefits. These include more accurate attention, higher spatial resolution in visual processing, enhanced mental rotation abilities, improved mood, increased relaxation, and lower levels of anxiety [13-15]. When used in moderation, video games can be a useful SEL tool for fostering positive behaviors and emotions. For instance, the Spanish video game *Aislados* was designed to improve youth psychological well-being and help them avoid risk behaviors related to addiction, violence, or emotional disorders. Those who played the game experienced increased health-related quality of life, positive affect, and mental health [13]. Additionally, research on the video game *Emociomente* showed that video games could promote emotional intelligence in adolescents when integrated with a guided and assisted framework [16]. Similarly, a systematic review highlighted the therapeutic and preventive benefits of video games in child and adolescent psychiatry [17], further supporting the potential of *Brain Agents* to address the needs of youth facing adversity.

Game-based learning has also been beneficial in clinical settings for mental health outcomes, provided there is a successful integration of health care and entertainment elements [18]. Research has demonstrated that games for mental health are generally accessible, feasible, and effective, supporting their potential to promote emotional and cognitive well-being in children and adolescents [19]. This aligns with the purpose of *Brain Agents* as a tool to foster resilience and SEL. *Brain Agents* was developed through an iterative process, incorporating student feedback to refine gameplay and enhance learning outcomes [20].

Video games, often viewed skeptically regarding their impact on child health, are favored leisure activities among children [21]. Engaging with games might help to develop emotional self-regulation skills, promoting mental health and well-being among children [11]. Besides emotional regulation, video games can also improve social skills [22]. This benefit supports the idea that game-based programs provide valuable opportunities to promote social-emotional competencies and adjustment in adolescence [23]. Research on prosocial video games highlights their ability to improve social outcomes in adolescence and emerging adulthood [24], consistent with *Brain Agents’* focus on fostering emotional and interpersonal skills. By leveraging the engaging nature of digital tools, such programs can spark children and adolescents’ interest in emotions and influence various personal, emotional, and social factors.

Moreover, the challenges in video games, such as facing failure and persisting through it, offer valuable life skills [25]. Persistence, analytical thinking, and self-efficacy are among the traits nurtured by engaging with challenging games, contributing to personal development. Overall, video games, when designed and used effectively, can positively impact child health, social development, and emotional regulation, presenting valuable opportunities for intervention and education.

### STRYV365 Interventions

Following principles from these studies, STRYV365, a nonprofit organization based in Milwaukee, Wisconsin, worked to advance and support the idea that educational video games could help children cope with ACEs. The goal was to be one of the first US studies to show that video games can improve SEL, resiliency, and positive life experiences in school children. STRYV365 partnered with the development studio Night City to design and develop the video game *Brain Agents*, with SEL and trauma-informed content as part of a multifaceted approach to foster resilience, promote mindfulness, and improve problem-solving and critical thinking in fifth to ninth-grade students. As others have found, we expect that when players participate and engage with *Brain Agents*, they can demonstrate openness, self-reflection, empathy, and promote tolerance of others’ perspectives [26].

For this block randomization study, in addition to providing *Brain Agents* to students in grades 5‐9 at 4 schools, the students also experienced the STRYV365 program *peak team*. The *peak team* coach mentor program strengthens students’ social-emotional skills and resilience through a trauma-informed approach integrating sports and mentorship, complemented by the *Brain Agents* video game. Grounded in principles of trauma-informed care, the program promotes emotional regulation, resilience, and decision-making by engaging students in physical activities such as teamwork-based games, reflective writing, and discussions. Delivered over 4 to 10 weeks in gym or physical education classes, the program combines fun and educational activities to empower students to overcome adversity and thrive. Qualitative research about *peak team* is reported in another paper.

The primary aim of this research is to describe the experiences of students participating in the study and their perceptions of the *Brain Agents* video game. The qualitative study uses the game to help students identify and describe their emotions, increasing awareness of their actions. This study also included an analysis of whether *Brain Agents* can be integrated into school settings to equip youth affected by trauma and adverse experiences with the skills and resilient mindset needed for a successful future. In surveys, focus groups, and interviews, the research team asked students to provide general feedback on the game, describe their personal experiences with *Brain Agents*, and offer suggestions for improving future versions of the game.

## Methods

### *Brain Agents* Game Design and Objectives

*Brain Agents*, created in collaboration between STRYV365, development studio Night City, and behavioral health professionals, was designed to foster resilience and support SEL by translating STRYV365’s established curriculum to an engaging digital game format. Targeting ages 10-15 years, the game uses narrative context and minigames to deliver SEL content, including trauma resilience, coping strategies, cognitive reframing, communication, relationship building, and emotional awareness. The game also incorporates STRYV365’s CRAFT process: Catch Yourself, Relax, Assess, Focus, Think, Decide, and Act.

### Development Process

Development of the game initiated in the spring of 2020 and has involved Columbia College Chicago game design and programming students, hired as interns through the US Federal Work-Study program. *Brain Agents* was developed using the Unity game engine and initially designed for mobile devices. However, due to Chromebook use in participating schools, the game was adapted for this platform. The game’s low polygon 3D art style ensures compatibility with lower-powered hardware while maintaining immersion and enjoyment from contemporary 3D game design.

### Early Access Model

*Brain Agents* uses an “early access” model, allowing students to play while the game is still in development. This approach can attract students interested in game development but may disappoint those expecting a finished product. Continuous, iterative improvements and content additions mean players’ experiences can vary, with later participants likely having a more refined experience. The development team has balanced student desires for features from popular games with the need to incorporate a learning agenda and the scope limitations of a nonprofit indie game project.

### Access and Future Availability

As of publication, *Brain Agents* is not publicly available. Study participants access the game via a designated website using a special code name. Future plans include making the game available in schools, youth organization programs, and potentially at home to support parents.

### Narrative Context and Gameplay Mechanics

**Figure 1.**
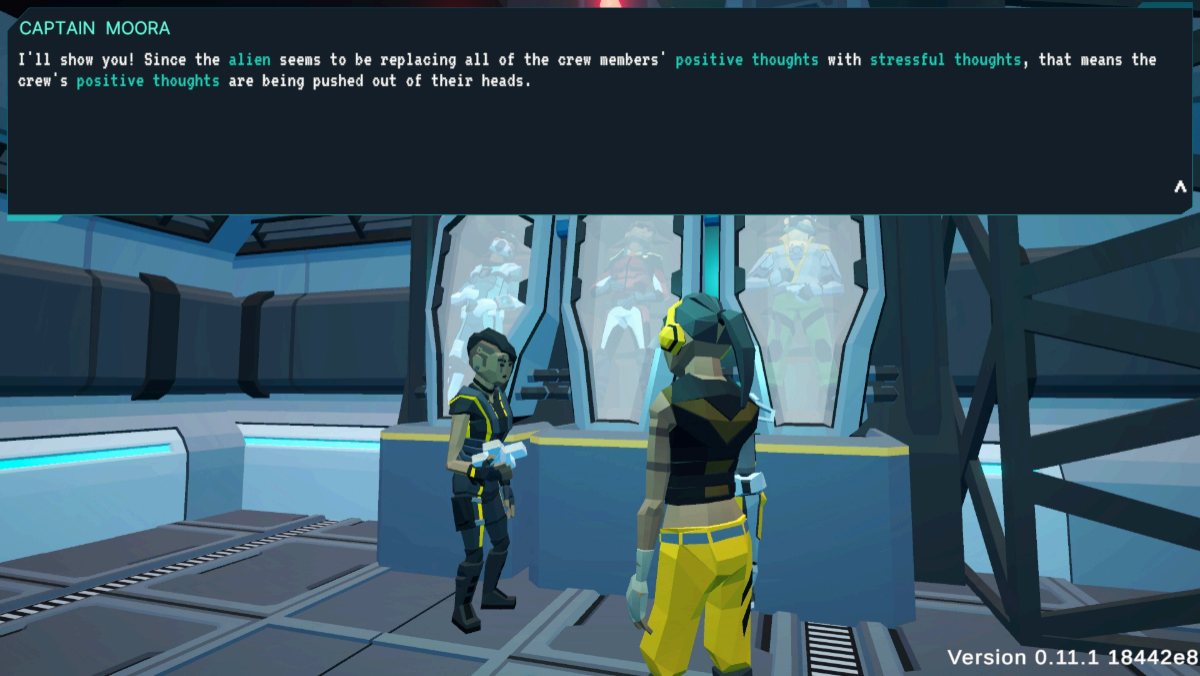
*Brain Agents* gameplay showing narrative context. The ship captain explains that the cryo-sleeping crew’s positive thoughts have been displaced by an alien computer virus that feeds off chaos and negativity. The displaced positive thoughts have been scattered around the ship in the form of “positive energy collectibles” that the player must find and collect.

In *Brain Agents*, players are explorers on a space vessel, responding to a distress signal. They encounter an alien computer program named MAL that infects both computer systems and human minds, causing cognitive distortions in the crew. Players, guided by the ship’s captain, must gather positive energy to rescue the crew by reframing these cognitive distortions (Figures1  2).

**Figure 2.**
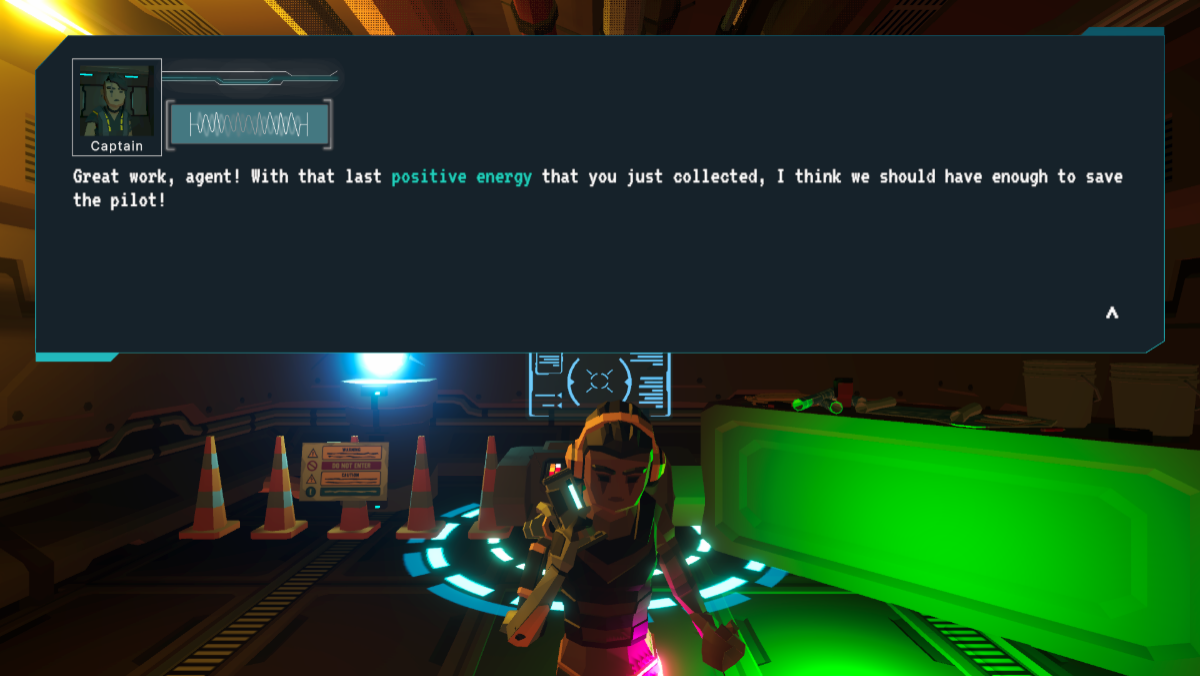
*Brain Agents* gameplay showing energy collection via positive thought construction. Upon gathering enough positive energy, the player attempts to save a crew member from the alien computer virus.

### Minigames and Learning Objectives

During this study, *Brain Agents* included 5 minigames that help players practice coping mechanisms, connect with emotions, and build social relationships with nonplayer characters. After this study, additional minigames have been developed.

**Figure 3.**
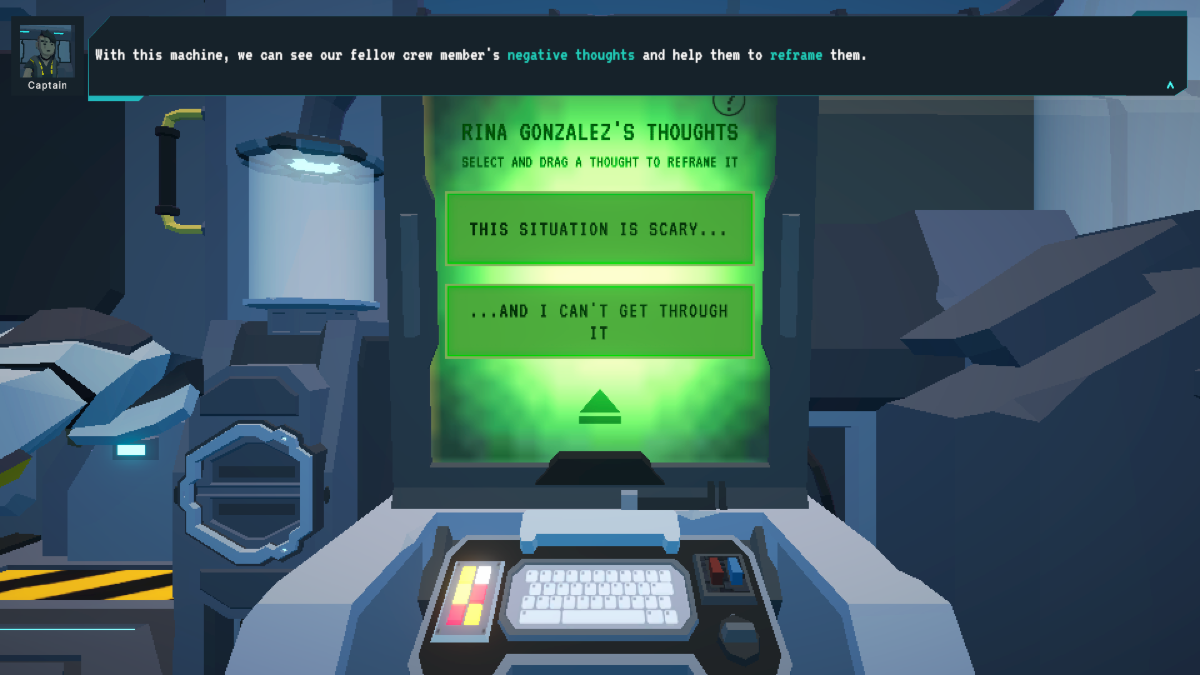
*Brain Agents* reframing minigame. To rescue members, the player deposits collected positive energy into a “reframing machine” that shows a cognitive distortion within a slumbering crew member’s mind. Each thought is divided into 2 sections, and the player must identify which is an unhelpful cognitive distortion and eject it.

Of the reframing minigame, players deposit positive energy into a reframing machine to transform cognitive distortions into constructive thoughts, rescuing crew members from MAL’s influence (Figures3  4).

**Figure 4.**
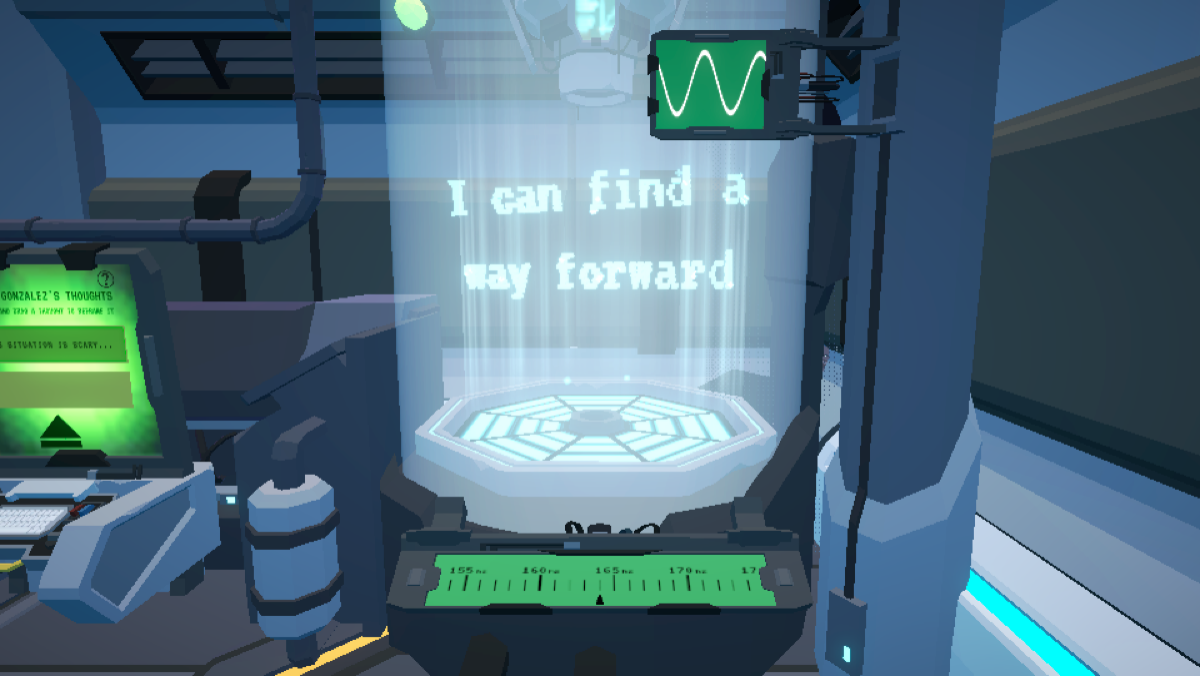
*Brain Agents* reframed positive phrases from the reframing minigame. Upon identifying the unhelpful thought to reframe, the player must adjust a dial to the “sweet spot” to reframe it into a constructive, positive idea.

Of the breathing minigame, players practice controlled breathing to calm themselves after in-game challenges, reinforcing mindfulness and relaxation techniques.

**Figure 5.**
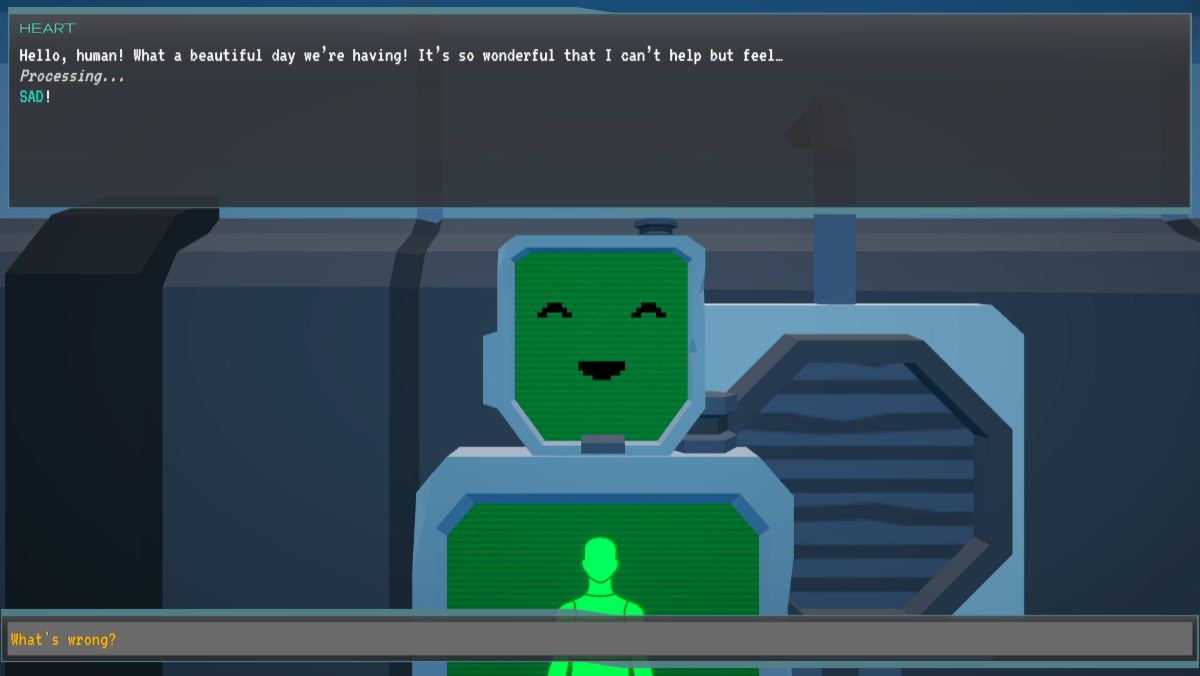
*Brain Agents* character HEART from the body chart minigame for identifying the physical manifestation of emotions is accessed through a robot named HEART that needs the player’s help in learning about human emotions. HEART: Human Emotion Acquisition Robot.

Of the body chart minigame, players identify physical manifestations of emotions on a body chart and associate colors with feelings, helping them become more attuned to their emotions (Figures5  6).

**Figure 6.**
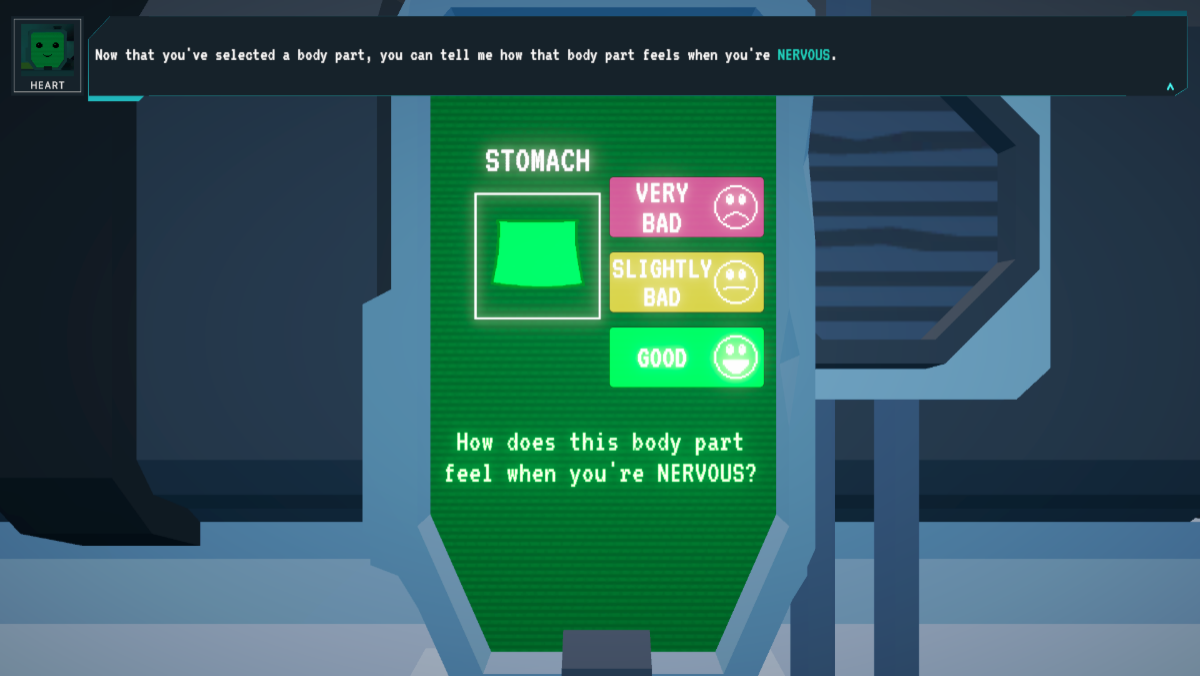
*Brain Agents* body chart minigame HEART asks the player to identify how their physical body feels when they experience various emotions. HEART: Human Emotion Acquisition Robot.

**Figure 7.**
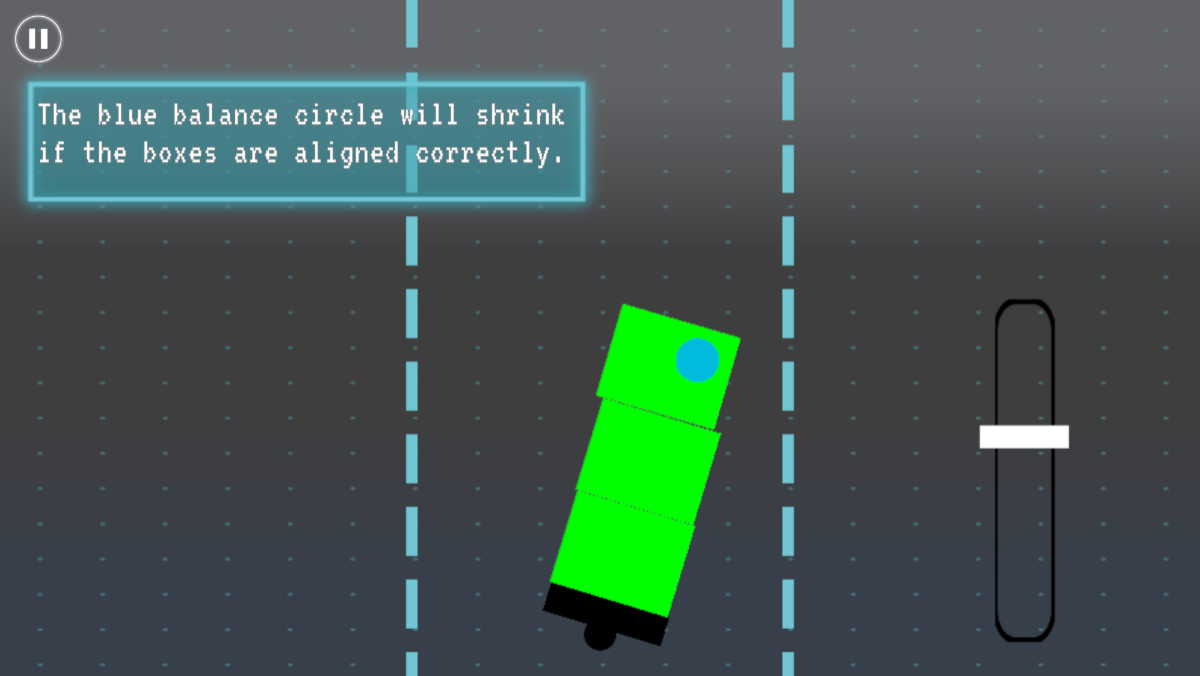
*Brain Agents* balance minigame. The player must catch blocks and lean a tower without tipping it over. The minigame uses gyroscope motion input to capture physical movement on devices equipped with one.

Of balance minigame, players balance a tower of blocks to build a firewall, emphasizing calmness under pressure and teamwork (Figure 7).

**Figure 8.**
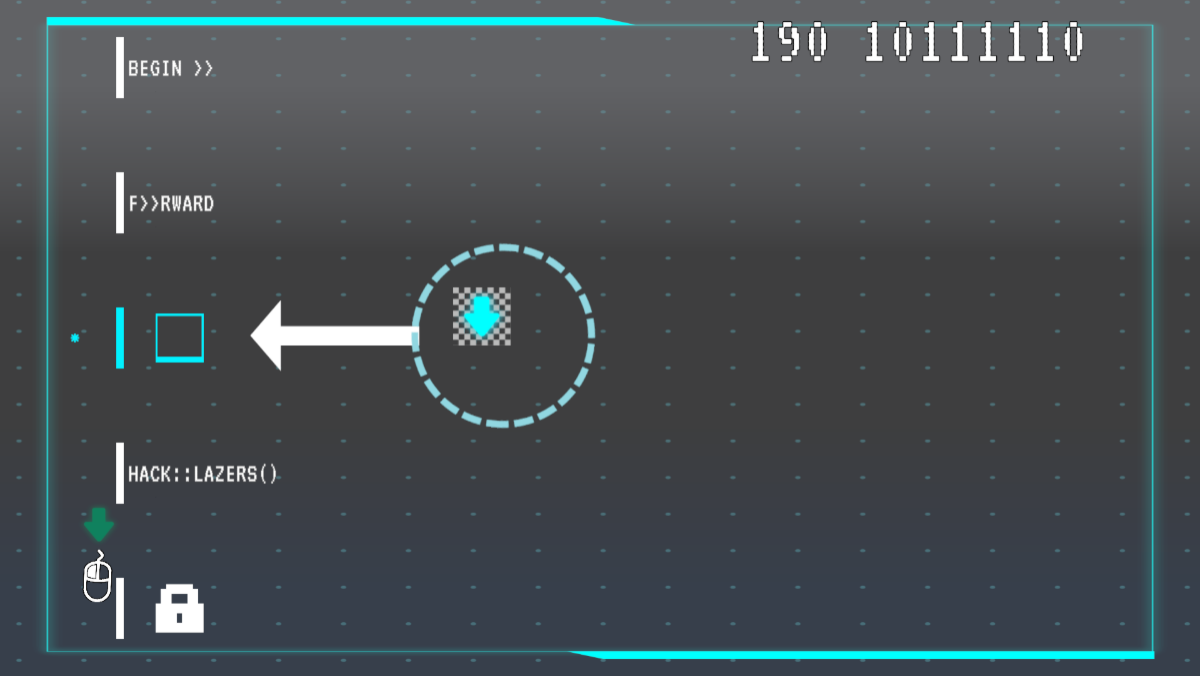
*Brain Agents* hacking minigame. Players must rearrange directional command blocks to create a path from the beginning to the goal. This image shows the tutorial where the simplest puzzle is presented to teach how to play.

Of the hacking minigame, players solve logic puzzles, reinforcing problem-solving skills and agency through the theme that systems can be changed and improved (Figure 8).

### Ethical Considerations

The Medical College of Wisconsin Human Research and Review Board (PRO037500) approved the research protocol for this evaluation. Parents and caregivers consented for their eligible children to participate in the research, students assented to their participation, and teachers and school administrators consented to joining focus groups. Privacy and confidentiality were protected throughout this study. Students provided their identity in surveys to link the data longitudinally over 2 years, then the data were deidentified for analyses. Students and teachers were deidentified in qualitative data analysis. Participants were not compensated.

### School Study Setting

STRYV365 partnered with 4 Milwaukee schools (2 public and 2 private choice schools), involving 1626 students in grades 5‐9. The demographic breakdown from school data was 991 (60.9%) Black or African American, 309 (19.0%) White, 179 (11.0%) Hispanic or Latinx, 98 (6.0%) Asian or Hmong, and 49 (3.0%) mixed racial or ethnic backgrounds, with 1122 (69.0%) experiencing economic disadvantage. In state standardized examinations, 943 (58.0%) were below basic, 439 (27.0%) basic, and 244 (15.0%) proficient in English, language arts, and mathematics. A total of 455 (28.0%) of students were chronically absent. Despite these challenges, 1431 (88.0%) of students at these schools earned high school diplomas when the study began, indicating a strong commitment to education and resilience among the student population.

### Researcher Qualifications and Relationships (Reflexivity)

This study was conducted from a positivist paradigm, using sociocultural theory to inform the conceptual framework. This approach allowed for the coconstruction of data and analysis through participants’ and researchers’ voices. The research team included members with similar racial or ethnic backgrounds and traumatic experiences as the participants, enhancing reflexivity. The coauthors are professionals in the health and education fields.

### Sampling, Recruitment, Consent, and Assent

CITI (Collaborative Institutional Training Initiative)–trained STRYV365 and school staff recruited participants from a convenience sample of 1626 students’ parents or caregivers, who provided informed consent digitally. A video at the STRYV365 website [27] summarized the consent form. Parents or caregivers were recruited at back-to-school events, open houses, parent-teacher conferences, and through emails in education tools PowerSchool, Google Classroom, ClassDojo, and school dashboards. Students provided written assent in REDCap (Research Electronic Data Capture; Vanderbilt University) before completing surveys and participating in focus groups or interviews.

Children were selected for focus groups and interviews based on their participation in the *peak team* and *Brain Agents* programs during the preceding semester. The randomized selection aimed to ensure a diverse representation of students in terms of grade level, gender, and race or ethnicity. Focus groups and interviews of students were conducted in a comfortable and confidential setting. Focus groups with teachers and school administrators were done in person or via videoconferencing. The findings have been summarized and discussed with parents or caregivers and students at the end of this study.

### Conceptual Framework and Study Design

This study hypothesizes that *Brain Agents* will positively impact student feelings, attitudes, behaviors, and school performance. A pilot cross-sectional series of surveys and focus groups was conducted in the 2021-2022 academic year [28], followed by a cluster randomized, incomplete block factorial, crossover design in the 2022-2024 academic years (Multimedia Appendix 1). Surveys were conducted at 6 time points, with key outcomes to be reported in 2025 (Multimedia Appendix 2).

### Data Collection

Baseline surveys were completed in October-November 2022, with additional surveys and focus groups or interviews in January-February and April-May 2023. Research staff facilitated focus groups with younger students and individual interviews with older students, ensuring comfort and openness. Audio recordings were transcribed for analysis.

### Qualitative Methods

Qualitative data were collected through focus groups and interviews conducted at the end of the fall and spring semesters to evaluate both *Brain Agents* and *peak team* programs’ impact on students’ SEL outcomes. Research staff facilitated focus groups with fifth to seventh graders, which encouraged peer interaction and group discussion dynamics, while individual interviews with eighth to ninth graders allowed for more in-depth exploration of personal experiences and perspectives. This approach ensured both comfort and openness for participants across age groups.

The qualitative evaluation was guided by a semistructured interview protocol (Multimedia Appendix 3), designed to elicit both open-ended responses and targeted insights aligned with the study objectives. Audio recordings of all focus groups and interviews were professionally transcribed and securely stored to maintain confidentiality.

Data processing and analysis were conducted using Dedoose (Sociocultural Research Consultants, LLC) software. A thematic analysis approach was used, involving an iterative process of generating and refining codes and themes. Coding began inductively, allowing themes to emerge naturally from the data, with additional subthemes identified during subsequent analysis.

A team of 7 coders participated in the analysis, with 2 coders independently reviewing each transcript. Discrepancies in coding were resolved through discussion to optimize intercoder reliability and enhance the rigor of the analysis. This systematic approach ensured a comprehensive understanding of the qualitative data, with a focus on identifying patterns and themes related to the interventions’ impact on students’ SEL outcomes. Qualitative findings focused on teachers’ perspectives [29] and students’ views of *peak team* are published elsewhere.

## Results

### Participants

A total of 329 parents or caregivers consented to the participation of their children in this study and children assented to join this study. The demographics of 321 children included 132 (41.1%) girls, 170 (53.0%) boys, 10 (3.1%) nonbinary, 6 (1.9%) who preferred not to answer, and 3 (0.9%) other. Their reported race or ethnicity was 189 (58.9%) Black or African American, 112 (34.9%) White, 32 (10.0%) Hispanic or Latino, 22 (6.9%) Asian, 16 (5.0%) American Indian, and 6 (1.9%) Pacific Islander; the total exceeds 321 due to mixed race/ethnicity children. As of the winter of the second year of this study (2023), nearly 32/329 (9.7%) of students had dropped out, primarily due to leaving the school or parental withdrawal of consent.

### Qualitative Data Collection

Focus groups and interviews were conducted in private rooms at the schools. One-third of participants, equally split between boys and girls, who were exposed to *Brain Agents* during the 2-year study, participated in a focus group or interview about their experiences. Twenty-two students from grades 5‐7, aged 10‐12 years, participated in 1 of 6 focus groups, each lasting an average of 26 minutes. Forty students from grades 8‐9, aged 13‐15 years, participated in individual interviews, each averaging 22 minutes.

### Baseline Survey Results

Using SAS (IBM Corp) to calculate percentages, the baseline survey of 277 students revealed that 202 (72.9%) of students had someone close to them die, 147 (53.1%) had someone close in jail, and 80 (28.9%) had someone close with drinking or drug problems. Additionally, 39 (14.1%) felt nervous or anxious daily, and 33 (11.9%) felt down or depressed daily; school psychologists or counselors were notified to follow up with their families. Strengths and assets reported included 230 (83.0%) being satisfied with life and aware of their feelings, 183 (66.1%) able to calm down when upset, 175 (63.2%) having no physical fights in the past 3 months, and 199 (71.8%) talking with friends about problems. Additional survey findings are reported in another paper.

### Game Use

During year 1 (2022-2023), a total of 328 children enrolled in the research played *Brain Agents* at school only. These students were distributed as 98 (29.9%) in grades 5-6, 145 (44.2%) in grades 7-8, and 85 (25.9%) in grade 9. Students played the game 2 to 3 days weekly for 4 to 5 weeks, averaging 10 sessions per semester and 23 minutes per session, totaling approximately 3.8 hours of playtime. Most students did not complete the entire game.

In *Brain Agents,* players aim to rescue all 15 crew members and defeat the alien malware by collecting up to 5 positive energies in each of the 30 playable missions or levels. Players start with 3 missions unlocked. New missions can be unlocked by earning up to 3 stars per mission: 1 for completing the mission, 1 for finding all 5 positive energies, and 1 for completing the mission under a target time. Thus, the amount of positive energy collected and the number of stars earned reflect player success and progress in the game.

Game performance data, analyzed with statistical software SAS, showed that grades 5‐6 collected the most positive energy at the 75th percentile, grades 7‐8 had the highest median positive energy of 8 (IQR 3-16), and grade 9 had the lowest performance in all measures (Table 1). The association of game performance with individual survey, focus group, and interview responses is pending further analysis.

**Table 1.** *Brain Agents’* game performance by grade after 4 semesters of play.

	Participants, n	Minimum	Maximum	Median (IQR)
Overall				
Missions completed	328	0	13	3 (1.5-4)
Stars awarded	328	0	38	4 (2-6)
Positive energy	328	0	67	7 (3-14)
Crew rescued	328	1	9	2 (1-2.5)
Grades 5‐6				
Missions completed	98	0	13	2 (2-4)
Stars awarded	98	0	25	4 (2-6)
Positive energy	98	0	63	7 (4-12)
Crew rescued	98	1	9	2 (1-4)
Grades 7‐8				
Missions completed	145	0	13	3 (2-5)
Stars awarded	145	0	38	4 (2-7)
Positive energy	145	0	67	8 (3-16)
Crew rescued	145	1	9	2 (1-3)
Grade 9				
Missions completed	57	0	8	2 (1-4)
Stars awarded	57	0	17	3 (1-4)
Positive energy	57	0	42	6 (2-11)
Crew rescued	57	1	7	2 (1-2)

### Synthesis of Themes

Eight core themes emerged from student focus groups and interviews: experience with *Brain Agents,* awareness, and ability to control emotions, goals, current challenges, and perceptions of the neighborhood (Table 2). Themes were derived from specific research questions about life experiences and goals, SEL, resilience, and game evaluation.

**Table 2.** Themes and subthemes used to code transcripts of focus groups and interviews of participating students in year 1.

Theme	Subtheme
Experience with *Brain Agents*	Positive experience with *Brain Agents* Negative experience with *Brain Agents* Neutral *Brain Agents* experience How *Brain Agents* was not helpful Feedback on improving *Brain Agents*
Experience with *peak team*	Positive experience with *peak team* Negative experience with *peak team* Neutral *peak team* experience How to improve *peak team*
Qualities of most pride	Extrinsic qualities (good at sport, etc) Intrinsic qualities (perseverance, etc) Unable to name quality (negative self-image)
Emotion associated with loss of control	Anger Annoyance Stress or anxiety Positive reactions Suppress emotions No emotion Other
Coping strategies	Ask for help Perseverance or grit Self-calm Escape, distraction, or sleep Self-improvement, self-efficacy, or choices Time management Retaliation No coping strategies
Future goal in 5 years	Educational goal Career goal Stability goal Extracurricular activities or hobbies Other goal No goal
Challenges in life	Family challenges School challenges School focus Sports or extracurricular challenges Anxiety Depression New responsibilities
Neighborhood or neighbors	Peaceful or calm neighborhood Loud or noisy neighborhood Rough neighborhood Busy neighborhood Can rely on neighbors Cannot rely on neighbors Feel safe in neighborhood Feel unsafe in neighborhood

#### Experiences With *Brain Agents*

*Brain Agents* was generally described as fun and helpful, particularly in improving self-awareness, focus, and emotional regulation. Positive feedback included comments on its calming effects and its role in enhancing critical thinking and problem-solving skills. Some students found it boring or had trouble with gameplay, but overall, the sentiment was positive.

#### Suggestions to Improve *Brain Agents*

Students suggested improvements such as better graphics, customizable avatars, cooperative play, clearer instructions, more problem-solving opportunities, expanded maps, additional missions, and difficulty adjustments. They also recommended incorporating self-reflection before gameplay.

#### Neighborhood Relationships

Students generally felt safe in their neighborhoods and trusted their neighbors, although some reported neighborhood problems. Descriptions ranged from “quiet” and “safe” to “loud” with frequent emergency responses and occasional gunshots. A few students mentioned living in “rough” neighborhoods with violence, shootings, and break-ins. Most students, however, felt they could rely on their neighbors for safety and support.

#### Qualities of Most Pride

Students expressed pride in various traits, both intrinsic (eg, work ethic, determination, intelligence, and creativity) and extrinsic (eg, athleticism, artistic skills, and relationships). However, some students had negative views of themselves, with 1 student explicitly stating they had nothing to be proud of. Despite these few negative outlooks, most students could identify positive qualities.

#### Challenges in Life

Students discussed family challenges, academic focus, and new responsibilities as significant challenges. Some described dealing with family losses, while others focused on academic pressures like procrastination and anxiety about school projects. Older students mentioned balancing home responsibilities with school demands.

#### Emotions Associated With Loss of Control

Anger was the most frequently reported emotion leading to loss of control, often triggered by video game frustration or sibling annoyances. Some students reported trying to suppress their emotions to avoid negative outcomes, while others described actively trying to calm themselves.

#### Coping Strategies

Students reported various coping strategies, including self-calming, self-improvement, avoidance, asking for help, and perseverance. *Brain Agents* encouraged students to remain calm, consider options, and breathe when stressed, which some students mentioned as helpful in managing their emotions.

#### Future Goals

Students’ goals for the next 5 years varied but included themes of education, career aspirations, extracurricular activities, and personal ambitions. Educational goals included attending college, pursuing specific degrees, and finishing high school. Career aspirations ranged from engineering and medicine to real estate and entrepreneurship. Extracurricular goals involved sports, the arts, and club participation. Other personal goals included caring for family, obtaining a driver’s license, and living independently.

## Discussion

### Summary of Main Findings

This study used a qualitative design with triangulation of focus groups, interviews, and surveys to conduct an in-depth exploration of students' experiences with the *Brain Agents* game and its impact on their learning, resilience, and life experiences. After 1 year of STRYV365 *Brain Agents* gameplay, qualitative insights highlighted both challenges and strengths for participating youth. These preliminary results will guide future coaching and game improvements.

The research aimed to gather qualitative insights about student experiences with the *Brain Agents* video game and its ability to promote SEL in schools. Overall, students had a generally positive experience with *Brain Agents*, reporting improvements in self-awareness, focus, and critical thinking skills. Many found *Brain Agents* entertaining, although some reported negative experiences, including glitches that caused frustration or boredom. Younger students (grades 5‐6) found the game more challenging compared to older students (grades 8‐9), who had lower engagement and scores, indicating the game may be more effective for younger audiences. Suggestions for future improvements included better graphics, customizable avatars, clearer instructions, and appropriate difficulty levels.

The game-based SEL of *Brain Agents* had a positive impact on students, demonstrating its potential as a tool to promote SEL in schools. This study highlighted the prevalence of ACEs among students and showed that *Brain Agents* could be used to build coping skills and resilience in a fun and competitive environment. Teachers, parents, and students can use the game’s lessons both in school and at home to foster resilience and teach SEL skills. For researchers and game designers, this study highlighted both successes and areas for improvement in the game, emphasizing the potential of game-based learning in education.

### Comparison With Other Educational Games

The *Brain Agents* game shares similarities with other educational games such as the Spanish *Aislados* and Italian *Emociomente*. The *Aislados* study reported increases in health-related quality of life, positive affect, and mental health but no improvement in emotional intelligence [13]. While this study did not directly measure quality of life or mental health, many students reported positive experiences and joyful emotions after playing *Brain Agents*, suggesting potential improvements in these areas. Positive life experiences are protective against ACEs. Future survey data may provide more concrete evidence of improved mental health.

The *Emociomente* study linked their game to improved emotional intelligence [16]. Similarly, students reported increased awareness of emotions, use of coping strategies, and improved behavioral responses after playing *Brain Agents*. Techniques such as breathing exercises and self-calming taught in the game were applied by students in real-life situations, demonstrating the game’s practical impact on emotional regulation.

The potential of video games as therapy extends beyond educational settings, with a research agenda proposed by Carras et al [30], highlighting the value of commercial video games in therapeutic contexts. This underscores the broader applicability of *Brain Agents* as a tool for fostering resilience and emotional intelligence.

### Transferability of *Brain Agents*

*Brain Agents,* accessible online through personal or school computers and mobile devices, has high potential for dissemination to other schools and communities. Studies examining serious games for children with specific conditions, such as ADHD, have identified subgroup variations in effectiveness [31]. Insights from such research could inform future adaptations of *Brain Agents* to maximize its impact across diverse student populations. Currently offered only in English, the game could be developed further and translated to make a global impact, promoting youth coping and resilience worldwide.

### Limitations

A limitation of this preliminary study is the lack of complete objective survey measures as the primary outcome to assess students’ SEL, resilience, and positive life experiences. The survey results will be reported after the 2-year study is completed. The nonspecific qualitative insights from students who participated in both *Brain Agents* and other STRYV365 programs may not solely reflect their experiences with the game. Additionally, the diverse student population in 4 Milwaukee schools may not be generalizable to children in other settings. For example, systematic reviews of cognitive training interventions for autism spectrum disorder [32] suggest that similar game-based approaches may be tailored to support other neurodiverse populations.

In some cases, individual student game performance tracking was unreliable due to issues such as students sharing accounts and leaving the game running while not actively playing. These issues affected the measurement of average playtime and other game metrics. In year 2, the research team enforced the use of unique accounts for each student and encouraged proper game usage to ensure accurate data collection.

The qualitative nature of focus group and interview analyses also introduces potential bias. Although each transcript was coded by at least 2 individuals to minimize bias, the qualitative analysis process has inherent limitations due to human factors.

### Conclusions

This study suggests that a digital video game intervention can positively impact SEL skills, resilience, and behaviors such as coping skills. While similar studies have been conducted in Europe, this is one of the first of its kind in the United States. This study’s inclusion of students from grades 5 to 9 provides unique insights into the responsiveness of different age groups to SEL video games, with younger students appearing more engaged with *Brain Agents*. This study also captured experiences unique to marginalized children in urban schools, demonstrating that video games can be a useful tool in SEL education and building resilience and positive life experiences.

By the end of the 2023‐2024 academic year, students completed 2 years of surveys. The STRYV365 research team expects that these surveys, combined with school record analysis, will demonstrate the program’s impact on students’ SEL, resilience, and positive life experiences. In the next year, *Brain Agents* will undergo several updates, including a new logo, more varied and engaging mission content, refined target times for missions, additional minigames, deeper dialogue choices with crew members, and in-game customization options.

To enhance teacher experience, STRYV365 will work to better connect the game’s learning scenarios to classroom curricula, allowing teachers to reinforce concepts more effectively. From a research perspective, the game will implement improved performance tracking. Ongoing studies of *Brain Agents* will continue to use student feedback to refine and enhance the game, aiming to improve student SEL, resilience, and positive life experiences further.

## Supplementary material

10.2196/67550 Multimedia Appendix 1 Incomplete block factorial design for academic years 1 and 2.

10.2196/67550 Multimedia Appendix 2 STRYV365 student survey instrument.

10.2196/67550 Multimedia Appendix 3 STRYV365 guide for conducting focus groups and interviews.

## Abbreviations

ACE: adverse childhood experience
CITI: Collaborative Institutional Training Initiative
CRAFT: Catch Yourself, Relax, Assess, Focus, Think, Decide, and Act
REDCap: Research Electronic Data Capture
SEL: social-emotional learning
