# Supplementary material for: Game-Based Social-Emotional Learning for Youth: School-Based Qualitative Analysis of Brain Agents
Source: JMIR Form Res. 2025 Jul 24;9:e67550. doi: 10.2196/67550 (PMC12289224; doi:10.2196/67550)

Time 1 = early fall; 2 = after fall semester; 3 = after spring semester; BA = Brain Agents; PT = peak team; B = both BA and PT; C = control group; X = no participation


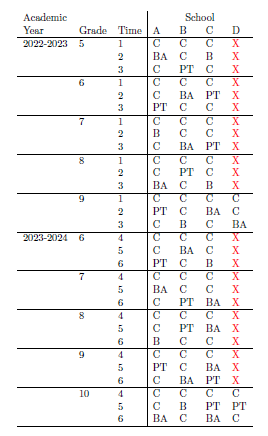

Supplement: Multimedia Appendix 1 [file formative-v9-e67550-s001.docx]
