# Supplementary material for: Game-Based Social-Emotional Learning for Youth: School-Based Qualitative Analysis of Brain Agents
Source: JMIR Form Res. 2025 Jul 24;9:e67550. doi: 10.2196/67550 (PMC12289224; doi:10.2196/67550)
Supplement: Multimedia Appendix 2 [file formative-v9-e67550-s002.pdf]

Scholar Questionnaire Winter 2024

Please complete the following survey as best as you can. You do not have to answer any questions that make you feel uncomfortable. You may ask for help from a teacher or coach.

What is today's date? (Click "Today")

____

The following questions about your name, date of birth, grade, school and email are to help us track your survey responses over time. After your responses are linked, your information will be removed from the data for analysis.

What is your first name?

____

What is your last name?

____

What is your date of birth?

____

Your email address

____

Where do you attend school?

◯ Brown Deer School District
◯ Howard Fuller Collegiate Academy
◯ Milwaukee Academy of Science
◯ Deer Creek/St. Francis School District

What is your current grade level in school?

◯ 6th Grade
◯ 7th Grade
◯ 8th Grade
◯ 9th Grade
◯ 10th Grade

What is your gender identity?

◯ Boy/Man
◯ Girl/Woman
◯ Non-binary/Gender fluid/Gender queer/Intersex
◯ Prefer Not to answer
◯ Other

Specify "Other"

____

What race/ethnicity do you consider yourself? (Check ALL that apply)
(Check all that apply)

☐ White
☐ Black or African American
☐ Asian
☐ American Indian or Alaskan Native
☐ Native Hawaiian or Pacific Islander
☐ Hispanic or Latino

I am satisfied with life.

◯ Mostly true about me
◯ Somewhat true about me
◯ A little true about me
◯ Not true about me

My life has a clear sense of purpose.

◯ Mostly true about me
◯ Somewhat true about me
◯ A little true about me
◯ Not true about me

When dealing with a problem, I consider several options to handle it.

◯ Mostly true about me
◯ Somewhat true about me
◯ A little true about me
◯ Not true about me

I am aware of my feelings.

◯ Mostly true about me
◯ Somewhat true about me
◯ A little true about me
◯ Not true about me

I can calm myself down when I am upset.

◯ Mostly true about me
◯ Somewhat true about me
◯ A little true about me
◯ Not true about me

I can talk about my problems with my family.

◯ Mostly true about me
◯ Somewhat true about me
◯ A little true about me
◯ Not true about me

I can talk about my problems with my friends.

◯ Mostly true about me
◯ Somewhat true about me
◯ A little true about me
◯ Not true about me

People in my neighborhood can be trusted.

◯ Mostly true
◯ Somewhat true
◯ A little true
◯ Not true

How many times have you moved during the past 12 months?

◯ Have not moved in the past year
◯ 1-2 times
◯ 3-4 time
◯ More than 4 times

Did you ever have anyone close to you die?

◯ Yes
◯ No

Did you ever have anyone close to you go to jail?

◯ Yes
◯ No

Did you ever have anyone close to you drink or use drugs so often that it caused problems?

◯ Yes
◯ No

During the past 3 months, have you hit, slapped, or pushed other people or gotten into a physical fight with them?

◯ Yes
◯ No

During the past 3 months, have you bullied or harassed anyone at school, through texting, social media (such as TikTok, BeReal, Twitch, Discord, Snapchat, Instagram, etc.) or through gaming platforms?

◯ Yes
◯ No

Over the last 2 weeks, how often have you been bothered by feeling down, depressed, or hopeless?

◯ Not at all
◯ Several Days
◯ More then half the days
◯ Nearly every day

Over the last 2 weeks, how often have you been bothered by feeling nervous, anxious, or on edge?

◯ Not at all
◯ Several Days
◯ More then half the days
◯ Nearly every day

Over the last 2 weeks, how often have you texted or used social media/gaming platforms, such as TikTok, BeReal, Twitch, Discord, SnapChat, Instagram, etc.?

◯ Not at all
◯ Several Days
◯ More then half the days
◯ Nearly every day

Over the last 2 weeks, how often have you been bullied, called names, harassed, or abused through texting, social media or gaming?

◯ Not at all
◯ Several Days
◯ More then half the days
◯ Nearly every day

peak team Program

Please rate how helpful the peak team program has been for you in the following ways.

Feel happier

◯ Not at all helpful
◯ Not so helpful
◯ Somewhat helpful
◯ Very helpful
◯ Extremely helpful

Feel more optimistic

◯ Not at all helpful
◯ Not so helpful
◯ Somewhat helpful
◯ Very helpful
◯ Extremely helpful

Try new things

◯ Not at all helpful
◯ Not so helpful
◯ Somewhat helpful
◯ Very helpful
◯ Extremely helpful

More aware of my feelings

◯ Not at all helpful
◯ Not so helpful
◯ Somewhat helpful
◯ Very helpful
◯ Extremely helpful

Make better decisions

◯ Not at all helpful
◯ Not so helpful
◯ Somewhat helpful
◯ Very helpful
◯ Extremely helpful

Bounce back from setbacks

◯ Not at all helpful
◯ Not so helpful
◯ Somewhat helpful
◯ Very helpful
◯ Extremely helpful

Listen and follow directions better

◯ Not at all helpful
◯ Not so helpful
◯ Somewhat helpful
◯ Very helpful
◯ Extremely helpful

Better cope with stress

◯ Not at all helpful
◯ Not so helpful
◯ Somewhat helpful
◯ Very helpful
◯ Extremely helpful

Better control my anger

◯ Not at all helpful
◯ Not so helpful
◯ Somewhat helpful
◯ Very helpful
◯ Extremely helpful

Better resolve conflicts without fighting

◯ Not at all helpful
◯ Not so helpful
◯ Somewhat helpful
◯ Very helpful
◯ Extremely helpful

Better connect with family

◯ Not at all helpful
◯ Not so helpful
◯ Somewhat helpful
◯ Very helpful
◯ Extremely helpful

Better connect with friends

◯ Not at all helpful
◯ Not so helpful
◯ Somewhat helpful
◯ Very helpful
◯ Extremely helpful

Better connect with neighbors

◯ Not at all helpful
◯ Not so helpful
◯ Somewhat helpful
◯ Very helpful
◯ Extremely helpful

Write other ways that peak team helped you.

How can we make the peak team program better?

Brain Agent Game

Please rate how helpful the Brain Agent game has been for you in the following ways.

Feel happier

◯ Not at all helpful
◯ Not so helpful
◯ Somewhat helpful
◯ Very helpful
◯ Extremely helpful

Feel more optimistic

◯ Not at all helpful
◯ Not so helpful
◯ Somewhat helpful
◯ Very helpful
◯ Extremely helpful

Try new things

◯ Not at all helpful
◯ Not so helpful
◯ Somewhat helpful
◯ Very helpful
◯ Extremely helpful

More aware of my feelings

- ◯ Not at all helpful
- ◯ Not so helpful
- ◯ Somewhat helpful
- ◯ Very helpful
- ◯ Extremely helpful

Make better decisions

- ◯ Not at all helpful
- ◯ Not so helpful
- ◯ Somewhat helpful
- ◯ Very helpful
- ◯ Extremely helpful

Bounce back from setbacks

- ◯ Not at all helpful
- ◯ Not so helpful
- ◯ Somewhat helpful
- ◯ Very helpful
- ◯ Extremely helpful

Listen and follow directions better

- ◯ Not at all helpful
- ◯ Not so helpful
- ◯ Somewhat helpful
- ◯ Very helpful
- ◯ Extremely helpful

Better cope with stress

- ◯ Not at all helpful
- ◯ Not so helpful
- ◯ Somewhat helpful
- ◯ Very helpful
- ◯ Extremely helpful

Better control my anger

- ◯ Not at all helpful
- ◯ Not so helpful
- ◯ Somewhat helpful
- ◯ Very helpful
- ◯ Extremely helpful

Write other ways that the Brain Agents game helped you.

What are ways to make the Brain Agents game better?

When finished, please be sure to click SUBMIT below.
