# Supplementary material for: Game-Based Social-Emotional Learning for Youth: School-Based Qualitative Analysis of Brain Agents
Source: JMIR Form Res. 2025 Jul 24;9:e67550. doi: 10.2196/67550 (PMC12289224; doi:10.2196/67550)
Supplement: Multimedia Appendix 3 [file formative-v9-e67550-s003.docx]

**STUDENT QUESTIONS**

Students with parent consent and their assent may participate in a focus group in grades 5-7 or individual interview in grades 8-10.

**A. Questions about the STRYV365 programs**

1. How was the *peak team or Brain Agents* program most helpful to you?
   1. Probe: *social-emotional functioning*; How did they feel when participating? (possible responses may be: feel happier, feel more optimistic, try new things, more aware of feelings, make better decisions, bounce back from setbacks, listen and follow directions better, better cope with stress, better control anger, better resolve conflicts without fighting; better connect with family, friends, or neighbors)
   2. Follow-up questions: Sometimes, we are unaware that things are helpful to us, even though we may have learned something new. Can anyone tell me something new they’ve learned from participating in STRYV programs? How did you apply that new thing you learned in your everyday life?
2. How was the *peak team* or Brain Agents program NOT helpful to you?
   1. Probe: STRYV staff interactions, activities/lesson plan impressions, and reasons for lack of engagement, access to staff when needed.
   2. Follow-up questions: If a student says something is ‘boring’ or provides a negative reaction, ask what aspect specifically about the intervention was boring to them—do not ask how we can make it better—that is asked during the next question. However, if a student provides that information, it will be associated with Q.3 during the analysis, so it is ok.
3. How can we make the *peak team* program (or *Brain Agents* video game) better?
   1. Probe: length of program, time of day when program is offered, activities. What did you think of the small games and activities within *Brain Agents*? To increase engagement, can you think of any ways that as a player you could be “rewarded” or create incentive in the game? Including a leaderboard? Frequency of adding or being able to access new challenges during the game?
   2. Follow-up questions: If a student is still stuck on how to respond, ask them to consider what they thought about the player avatar, narrative and story within the game, and/or the visuals/graphics/art style in the game.
4. Please tell me a story about your experience with the peak *team or Brain Agents*.
   1. Probing for: anxiety; willingness to try new things; ability to transition from one activity to another with different staff; inspirational stories; making mental connections between behavior and consequences; ability to be resilient
   2. Follow-up questions: How did you feel about STRYV conducting gym class rather than your regular teacher? If you participated in BA instead, how did it feel to have a STRYV coach in class asking you to play the game, rather than your teacher?
5. What else would you like to say about *peak team or Brain Agents*?

**Facilitator:** Thank you for sharing those details with us. I want to move on to another topic and ask about how you navigate some of life's challenges. Everyone experiences difficult times, and we are interested in how you handle stress and setbacks.

**B. Questions about social-emotional skills and resiliency**

1. What are some qualities about yourself that you're proud of?
   1. Probe for: *Subjective well-being.*
   2. Follow-up questions: You don’t have to be ‘good’ at sports or be especially creative to be proud of yourself for trying. Can you describe some aspects of your personality or some of your positive qualities or actions others may notice?

Think about a time when your emotions got the best of you. For instance, you lost your temper about something. (In the interest of time and allowing everyone to be able to answer, do not ask for situational details) For students who are able to answer on their own and do not need to be prompted for additional information, don't give any suggestions and just listen to their responses. However, for students that don't speak up, have to be prompt, or require further explanation, you can then provide them with both positive and negative examples.

1. Without providing specific details, what did you do to calm down?
   1. Probe for: *Self-awareness, self-management, and emotional regulation.*
   2. Follow-up: How long did it take for you to calm down? Was there someone in particular that helped you to do that? Who? *(The ‘who’ is important because it tells us about the trusted adults in their life).*
2. **INDIVIDUAL INTERVIEW QUESTION ONLY*:** Think of a specific challenge in your life. What are some things you do to help you overcome challenges in your life?
   1. Probe for: *Coping skills.*
   2. Follow-up questions: What is your "stress-reliever?" (You can provide examples for this one if students have a hard time answering): pray, exercise, alcohol, drugs, cut themselves on purpose to feel better, hang out with friends or neighbors who engage in potentially violent or illegal activity. How often do you do that? (Be cognizant of bias: do not ask them to choose if they think it is healthy or unhealthy behavior).

******Individual interviews will be conducted by a member of the research team and one note-taker****.* Suppose a student discloses any risk-taking behavior that will harm themselves or others. In that case, the school guidance counselor will be notified via confidential email from STRYV365, which must include a request to reply with an acknowledgment of receipt. Inform all participants that they may discuss their approach to stress relief with their parents, trusted teacher, or school counselor.

1. Close your eyes and take 10 seconds to imagine yourself five years from now. After time is up ask the group: What are you doing? What are some important decisions you may make to achieve your goals?
   1. Probe for: *Responsible decision-making skills.*
   2. Follow-up question: How long do you spend thinking about your future (every day? Every week? Never?)

10. Is there anything else you want to share with us today?

**INTERVIEW QUESTIONS ONLY**

11. Nearly half of US teens have been bullied, called names, harassed or abused through texting or social media or games such as TikTok, BeReal, Twitch, Discord, Snapchat, or Instagram. What’s been your experience with this online bullying?

a. Probe, if relevant: How have you tried to stop it?

12. Describe the neighborhood you live in. Include what it looks like but also tell me about your neighbors.

a. Probe for: *Social connectedness, community engagement, and support.*

b. Follow-up questions: Why does your neighborhood make you happy, sad, or scared to come home? Can your neighbors be trusted, or are you suspicious of them? Can you go to them for help when needed?

**TEACHER AND STAFF FOCUS GROUPS QUESTIONS**

1. What do you know about *peak team, Brain Agents* and the collaborative research study with STRYV365?
2. What are the strengths of STRYV365 programming?
3. After STRYV365 programs, what specific changes have you observed in student’s social interactions and emotional responses?
4. How useful has the STRVY365 training been in social emotional learning, trauma-informed care, and promoting positive life experiences?
5. What are memorable stories or narratives about students in STRYV 365 programming that you would like to share? We will remove any names or identifying information that is shared or reported.
6. How are your relationships with students, especially those who struggle with trauma and stress?
7. How do you feel about addressing student bullying, especially when it occurs through texting, social media, or gaming?
8. What impact have STRYV365 programs had on teacher and school staff recruitment and retention?
9. What are suggested opportunities to improve STRYV365 programming?
